# Analyzing the paradigmatic cases of two persons with a disorder of consciousness: reflections on the legal and ethical perspectives

**DOI:** 10.1186/s12910-021-00656-w

**Published:** 2021-07-08

**Authors:** Mario Picozzi, Lino Panzeri, Davide Torri, Davide Sattin

**Affiliations:** 1 Center for Clinical Ethics, Biotechnology and Life Sciences Department, University of Insubria, Varese, Italy; 2 Clinical and Experimental Medicine and Medical Humanities-PhD Program, Via Ottorino Rossi 9, 21100, Varese, Italy; 3 Department of Law, Economics and Cultures, University of Insubria, Via Sant’Abbondio 12, 22100 Como, Italy; 4 ASST Dei Sette Laghi - S.C. Medicina Legale, Viale Borri, 57, 21100 Varese, Italy; 5 Neurology Public Health and Disability Unit, Fondazione IRCCS Istituto Neurologico C. Besta, Via Celoria 11, 20133 Milan, Italy

**Keywords:** Disorders of consciousness, Vegetative state, Reconstruction of self-expression, Time of care, Relationship between law and ethics, Eluana Englaro, Theresa Marie Schiavo

## Abstract

**Background:**

Media have increasingly reported on the difficulties associated with end-of-life decision-making in patients with Disorders of Consciousness (DOC), contextualizing such dilemma in detailed accounts of the patient’s life. Two of the first stories debated in the scientific community were those related to the cases of two women, one American, the other Italian, who captured attention of millions of people in the first years of this third millennium.

**Methods:**

Much has been written about the challenges of surrogate decision-making for patients in DOC, but less has been written comparing these challenges across legal systems and cultures. In our paper, we propose a systematic analysis of the final legal documents written by the American and Italian Courts in relation to the two cases, developing our discussion around three areas: the level of certainty/reliability of diagnosis and prognosis, the reconstruction of self-expression, time of illness and time of care. They are examples of the typical issues discussed by legal authors and allow us to understand the link and the difference between the legal and ethical perspectives.

**Results:**

The legal approach to the two cases has some common elements: the need to be certain about the diagnosis and prognosis and the fact that the clinical criteria are necessary in determining the most appropriate treatments, although these criteria are not sufficient unless they are supplemented by the patient’s will. The issue of relations takes on importance both from a legal and an ethical point of view, but from two different perspectives. While ethics safeguards relationships by guaranteeing their differences and makes them reconcilable, law safeguards relationships by guaranteeing the cold forms of respect, equality, impartiality, symmetry, reciprocity, and irreversibility. In this perspective, the link between the time of care and the decision of the family members assumes importance.

**Conclusions:**

The most interesting point that emerges from our analysis is the issue of relationships and how they affect decisions, both from a legal and ethical point of view. For this reason, during the patients’ hospitalization, it is necessary to identify ways in which they might give their opinion about the moral issues underlying their choices.

## Background

Vegetative state (VS) is a clinical condition of wakefulness without awareness caused by traumatic and non-traumatic brain injuries [1]. The term “persistent vegetative state” was originally proposed by Jennett and Plum in1972 to describe patients with brain damage in a state that “was no longer coma but was not recovered and that did not demonstrate any adaptive response to the external environment” [2]. Patients in VS normally have their eyes open while awake and closed while asleep, they breathe spontaneously, have preserved autonomic function, eyes blink, have roving eye movements, and facial movements. They could have limb spasticity and pseudobulbar palsy. But to the fullest extent determinable, they lack awareness of themselves and their environment [3] and so they require complete assistance by other people.

The consensus statement built in 1990 by the American Medical Association's Council on Scientific Affairs and Council on Ethical and Judicial Affairs [4] presented the appearance of virtual unanimity among the governing elements of American medicine on a linked series of beliefs on the diagnosis, treatment, and ethical status of post-coma patients. These medical conventions are generally accepted unquestioningly as constituting a factual foundation for ethical debate on VS or unresponsive wakefulness syndrome (UWS), a condition that is still at the center of ethical debate, as demonstrated by the increasing number of articles published on it in the last years [5–7].

However, debate is not relegated to the the scientific platforms, but also amplified by media. In fact, media have increasingly reported on the difficulties associated with end-of-life decision-making in Disorders of consciousness (DOC), contextualizing such dilemma in detailed accounts of the patient’s life.

For example, the stories of Theresa Marie Schiavo (from now named TMS) [8, 9] and Eluana Englaro (EE) [10, 11], one American, the other Italian, captured attention of millions of people in the first ten years of this third millennium.

Patients with a Vegetative State diagnosis could be considered a paradigmatic case of extreme disability: they have a non-degenerative health condition, the impossibility to communicate with others and a high need of sustain from environmental factors [12].

Legal actions toward the end of life decisions were proposed by EE’s relatives in contrast to government policies and by TMS’s maternal family members who opposed TMS’ guardian decision. All judges involved in those legal processes required several medical evaluations but it is difficult to determine which and how much medical aspects (worst prognosis, incapacity to interact with the environment, absence of self-awareness, etc.) correlate mainly to a legal decision about the end-of-life of those patients. In addition, the evolutions of life decisions in time were studied, an analysis that could be useful for the future judgment and ethical debates on DOC.

Much has been written about the challenges of surrogate decision-making for patients in DOC, but these discussions are usually confined to a particular legal system. Less has been written comparing these challenges across legal systems and cultures. In our paper, we propose a systematic analysis of the final legal documents written by American and Italian Courts in relation to the two above mentioned cases to highlight themes requiring further ethical scrutiny. This analysis will be developed around three points: the level of certainty/reliability of diagnosis and prognosis, reconstruction of self-expression, time of illness and time of care. These points represent issues discussed in both ethical and legal literature.

## Methods

### Biographical sketch

#### Theresa Marie Schindler (Schiavo) (TMS)

Theresa Marie Schindler was born on the 3rd December 1963, and in the early morning of February 25 in 1990, she was taken to Humana North-side Hospital in St. Petersburg after a cardiac arrest. The cause of the collapse is disputed (probably caused by low potassium levels due to an eating disorder) and when Theresa Schiavo emerged from the coma she was in a completely unresponsive state in which she would remain for the next fifteen years (although it has been debated if she actually was in Minimally conscious state, as was sustained after the presentation of a video by media).

A ruling by Judge George Greer of the 6th Judicial Circuit Court in Clearwaterin in February 2000, based on the testimony of Michael Schiavo (husband), his brother, and his brother’s wife, in which was shown that Theresa made casual statements to them a year before her injury that she would not want to be kept alive artificially, established that Theresa’s feeding tubes (through the feeding tubes both hydration and nutrition are provided) must be removed. The Schindler family maintains Theresa has consistently exhibited a strong will to live, and they opposed stopping their daughter's nutrition. Judge Greer, in November 2002, reaffirmed the Court’s original ruling and he ordered the feeding tubes be pulled January 3, 2003. Numerous Courts of Appeal and federal Court motions delayed this second removal until October 15, 2003. TMS endured six days of dehydration before public support for the incapacitated woman prompted Florida’s Legislature to pass “Terri’s Law”, which empowered Florida’s Governor to order Theresa Schiavo’s feeding tubes reinserted. Some months later, after having passed on the opportunity to adjudicate other aspects of the Theresa Schiavo case, Florida’s Supreme Court bypassed the 2nd District Court of Appeal and assumed jurisdiction over the “Terri’s Law” appeal. The U.S. Supreme Court later paved the way for the third removal of Theresa’s feeding tube. TMS died in a hospice in Pinellas Park on March 31, 2005.

### Eluana Englaro

In January 1992, in Italy, Eluana Englaro was aged 21 when she had a road accident and she was admitted to hospital where an anoxic brain damage was found. Her father had fought for removing her feeding tube for over a decade because he claimed that Eluana specifically brought up the subject a year before her accident when a friend of hers had a motorbike accident and suffered severe brain damage. She had told her father that she would prefer to die and not be resuscitated if she were in such state. The Court of Appeal in Milan decided that Eluana’s condition was irreversible and that the deciding factor should be the patient’s own expressed wishes prior to the incapacity, as recalled by her family and friends. Finally, in 2008, Englaro won the support of the highest Court. The Court of Cassation decreed that feeding could be stopped [13]. After this decision, various organizations, religious and not religious, sought to have the Court’s ruling annulled by the European Court of Human Rights, but they were unsuccessful. Meanwhile, the Welfare minister, and a Vatican’s Spokesperson affirmed that this decision was wrong, and that withdrawal of artificial nutrition and hydration was an “inhuman murder”. Mr. Englaro decided to move her daughter to a private clinic in order to implement the ruling, given that the nursing home in which she had been since 1992 had opposed the implementation of such decision. The Ministry tried to declare the private clinic’s activity (in which Eluana was hospitalized after that Lombardy’s Authorities declared that any public clinic of its territory had not to stop nutrition and hydration in this particular case) as illegal, and threatened to push legislation through Parliament discussion. Eluana died three days after doctors of the private clinic began a medical protocol of a gradual withdrawal of nutrition and hydration.

### The grid for the legal documents’ analysis

An ad-hoc schedule was developed to analyse each document found.

The grid was composed of three areas. They represent the typical issues discussed in both ethical and legal literature (e.g. 14–17).

1. Level of certainty/reliability of diagnosis and prognosis:* in this part all the sentences referring to clinical diagnosis of patients and prognostic data have been reported or indicate, for example, to what extent diagnosis and prognosis were reliable.*
2. Reconstruction of self-expression: *in this cell was reported part of the text with* the role of the family members in reconstructing the will of the patients.
3. Time of illness and time of care:* we searched for sentences related to “time spend with” considering a wide range of arguments (e.g. time passed in VS, time of justice, lived time, chronological time, the role of time in decision-making, *etc*.).*

In the manuscript we also reported the legal references found in the text in order to allow readers to analyze all cited materials. Sources were selected considering the main stages of the legal proceedings of EE and TMS. The documents, which were studied in the original language, were downloaded from selected websites. As these are public materials, no consent was required.

All the documents were firstly analyzed by one rater (LP) who extracts the sentences from the analyzed material. All the found sentences were then discussed with the other two raters (MP and DS) for the final selection.

## Results

In the following paragraphs, we report in which way the three themes are being considered in the legal decision.

When found, we report the entire sentences in the text that refers to the dimensions analysed, whereas we describe/summarize the concepts we found in the two legal documents when we noted that the text could be open to interpretation regarding the topic, as there in no specific sentence.

## *The level of certainty/reliability of diagnosis and prognosis*:

The texts show how—from a legal point of view—it is necessary to have solid scientific evidence regarding the diagnosis and prognosis and how this has been a constant factor in both cases at all stages of the proceedings*.*

### TMS

Permanent vegetative state is

*a permanent and irreversible condition of unconsciousness in which there is: (a) The absence of voluntary action or cognitive behavior of any kind; (b) An inability to communicate or interact purposefully with the environment*.

*The medical evidence before this Court conclusively establishes that she [TMS] has no hope of ever regaining consciousness and therefore capacity, and that without the feeding tube she will die in seven or fourteen days” […] “the overwhelming credible evidence is that Terry Schiavo has been totally unresponsive since lapsing into the coma almost ten years ago, that her movements are reflexive and predicated on brain stem activity alone* […] (Judgment 11th of February 2000, so-called Schiavo I).

*The evidence is overwhelming that Theresa is in a permanent or persistent vegetative state” and “Unless an act of God, a true miracle, were to recreate her brain, Theresa will always remain in an unconscious, reflexive state, totally dependent upon others to feed her and care for her most private needs. She could remain in this state for many years* […] (Judgment 24th of January 2001—District Court of Appeal of Florida).

[*H]er actions were neither consistent nor reproducible but rather were random reflexes in response to stimuli*.

Finally the Court states that:

*viewing all of the evidence as a whole, and acknowledging that medicine is not a precise science, the Court finds that the credible evidence overwhelmingly supports the view that Terry Schiavo remains in a persistent vegetative state* […] *the real issue in this case, however, deals with treatment options for Terry Schiavo and whether or not they will have any positive effect so as to significantly improve her quality of life* […] *It would not appear from the testimony that this is a viable treatment option at this time* (Judgment 22nd of November 2002, Florida Sixth Judicial Circuit).

### EE

Treatments can only be interrupted if a PVS is diagnosed. It represents a condition in which,

*as a result of a rigorous clinical valuation, there is no medical basis, according to the internationally recognized scientific standards, that suggests that the person has even the slightest chance of some, albeit weak, recovery of consciousness* (Court of Cassation, Civil Section, Judgement No. 21748/2007).

In this case, judges ascertained that

*EE was able to breathe spontaneously, her cardiovascular, renal and gastrointestinal functions were preserved but she was unable to have cognitive and emotional experience and so to have a contact with external environment*.

Moreover,

*she did not show signs of psychical events and participation in environment activities, and no signs of voluntary behavioral responses to external visual, auditory, tactile and nociceptive stimuli*.

### Reconstruction of self-expression

#### TMS

The judgements specify that there is no written statement by the patient which makes it necessary for the proxy to reconstruct her will.

*Before exercising the incapacitated patient’s rights to select or decline health care, the proxy must comply with the pertinent provisions applicable to surrogates under this chapter, except that a proxy’s decision to withhold or withdraw life-prolonging procedures must be supported by clear and convincing evidence that the decision would have been the one the patient would have chosen had the patient been competent*.

The two opinions (of the husband and the parents) are highlighted in reconstructing and therefore interpreting Theresa’s thought. The husband argues

*that she related her feelings to an uncle of hers who was severely injured in an automobile accident and was comatose for a time. At one point, according to Mr. Schiavo, during a train trip from Pennsylvania to Florida in the mid-1980s, the ward told her husband that if she were ever in a situation of being artificially maintained she wanted the life support removed*.

*In cases of doubt, we must assume that a patient would choose to defend life in exercising his or her right of privacy*

The parents instead argue that

*the ward never discussed with them what her intentions would have been concerning the withholding or withdrawal of artificial life-prolonging procedures” and “they also have not knowing that the ward spoke of these matters with anyone else*.

#### EE

According to the Court of Cassation, the legal system provides that

*individuals who have made it clear, explicitly or through their opinions, lifestyle and values, before falling into a state of complete and absolute unconsciousness typical of a permanent vegetative state, that they would not accept the idea of their body outliving their mind thanks to a medical treatment*,

have the

*possibility to ‘have a say’ about interrupting the treatment through a legal representative* (Court of Cassation, Civil Section, Judgement No. 21748/2007).

Then the role of the legal representative is specified. It must be possible for this will to be expressed through a legal representative; however, it is specified that

*since it must be aimed at protecting the right to life of the represented party, the power of representation may allow for the treatment interruption only in exceptional circumstances*.

While acting

*in the best interest of the incapacitated persons*,

their legal representatives must make decisions

*neither in their stead or on their behalf but with them*, hence *reconstructing the alleged will of the unconscious patients, who were already adults before falling into that state, by taking into account the wishes they had expressed before losing consciousness*.

Their representatives’ petition must

*really express, based on clear, unambiguous and convincing evidence, the patients’ will […], deduced from their previous statements or personality, their lifestyle and beliefs, corresponding to their notion of dignity of the human person, before falling into the state of unconsciousness*.

Act No 833 of 23rd of December 1978, after premising in Section 1 that

*the protection of the physical and psychical health must go hand in hand with the respect of the dignity and freedom of the human person*,

establishes the usually voluntary nature of healthcare checks and treatments (Section 33).

### Time of illness and time of care

#### TMS

There was a reference that specified that TMS was

*not after a few weeks in a coma, but after ten years in a persistent vegetative state that has robbed her of most of her cerebrum and all but the most instinctive of neurological functions, with no hope of a medical care* […].

Theresa’s husband, her legal representative, submitted a formal request for the removal of the feeding tube in May 1998.

*It would be unrealistic to expect the proxy to pretend that the ward was not aging and remained twenty-five. The proxy had to use the best available evidence to ascertain the decision that Theresa Marie Schiavo would have made in February 2000 if she had remained competent to assess her own terminal condition and make her own informed decision* (Judgment 11th of July 2001, District Court of Appeal of Florida, Second District, so-called Schiavo II).

### EE

In the documents, “time” was used in relation to the long-duration of the vegetative state as a demonstration of the non-riversibility. The time needed for considering a patient in VS irreversible is considered to be three months for a child and one year for an adult (position affirmed by a neurologist during the legal process) which are shorter times than the number of days spent in VS by EE.

Formally, Eluana’s father, her legal representative, asked for suspension of hydration and nutrition in 1999, seven years after the accident.

From a legal point of view, both the American and Italian approaches show that the legal representative is considered as a witness, that is, the one who, thanks to his relationship with the patient, is in the best position to report his opinion. But, at the same time, it is highlighted how conflict arises exactly from the different interpretation that the people who live next to the patient give of his desires.

## Discussion

### Diagnosis and prognosis

The first point we want to discuss is related to the diagnosis and prognosis area. In both cases, the judges required several clinical evaluations to establish a correct diagnosis and the professionals seemed to be unanimous on to the diagnosis of Vegetative State both for TMS and EE. We think that all the clinicians involved in the assessment of the two cases thought carefully about their work and probably the diagnosis of VS was correct for the two women. However, we want to point out that it is challenging for clinicians to make a DOC diagnosis and real attention should be used when a profound knowledge of the pathology is still lacking [18]. Moreover, it is worth pointing out that TMS’s and EE’s cases happened before the introduction of the minimally conscious state (MCS) criteria and before the validation of the Coma Recovery Scale-Revised [19, 20], the actual clinical standard tools used to make the diagnosis nowadays.

Some articles published after 2009, the year of the EE’s death, reported that the rate between a diagnosis of VS and MCS was sometimes over the 40% [21, 22] and that instrumental techniques require time before the validation of standard protocols that can support the clinical determination of a conscious level in patients after a severe brain damage [23]. Mistakes during a diagnostic process are frequent in the clinical setting but in the case of pathologies already not well-known (and that involves one of the biggest human mysteries like consciousness generation) we think that medical aspects should be analysed really carefully (in especially referring to some sentences found in the documents like “unable to have cognitive and emotional experience”) and not focusing on external behavior only. Recent research has shown that the presence/absence of awareness in patients diagnosed as being in a vegetative state might be evaluated using functional neuroimaging, thereby providing a way of assessing awareness that does not rely only on external behavior [24, 25]. The definition of a universal protocol with standard tasks is far to be implemented across nations. Moreover, absence/presence of consciousness in a patient is still inferred mainly by observed behaviors only because the same concept of consciousness is now undetermined. Medical research is very committed to solve this problem but a lot of grey areas are still present, and the new findings have led to an increased diagnostic sensitivity but, on the other side, have led to increased doubts about the really absence of awareness in a person after a severe brain injury. Considering this new scientific information, judges should deeply consider the diagnostic and prognostic elements in the future in order to better identify the clinical grey zones and so thus using more critical sense regarding the certainty of the diagnosis. We recognize this is a difficult task for every judge, as Courts need certainty to be able to choose: the higher uncertainty level, the more cautious you need to be, especially when making irreversible decisions such as suspending treatment. At the same time, even those called upon to give voice to the patient must understand the congruity of the wishes previously expressed by the patient with his current clinical situation.

### Reconstruction of self-expression

Knowing the difficulty of analysing legal and ethical discussion on persons with DOC, we want to focus on one issue in our discussion: in both TMS’s and EE’s cases, the people that work for the care of the patients were the subjects who began the legal process. The patients were unable to do it by themselves of course, but we found particularly important that the judges were consulted when, and only when, there was a contrast between two parts (husband vs parents and father vs Italian State respectively) around two points (law as a guarantee for life and reconstruction of self-expression) and not for other things, like types of medical treatments or economic ones.

In the Italian Constitution, Section 32, Paragraph II, specifies that no one may be obliged to undergo a specific health treatment, except by legal provision, which cannot, in any case, violate the limits imposed by respect for the human person. Also at the European level, the Oviedo Convention (‘Convention for the Protection of Human Rights and Dignity of the Human Being with regard to the Applications of Biology and Medicine: Convention on Human Rights and Biomedicine’), adopted in Oviedo on 04.04.1997 and ratified by the Italian State with Law No. 145/2001, reaffirmed that “an intervention in the health field may only be carried out after the person has given free and informed consent to it” and that “the person concerned may freely withdraw consent at any time” (Section 5).

These decisions include both objective and subjective clinical criteria, such as the quality of life. In this perspective, where the person is no longer able to express himself/herself, the guardian appointed to protect his/her interests should witness the patient’s will (it is worth reminding that in Italy, at that time, there was no legal recognition of the advanced directives later regulated by Law No. 219/2017). When these elements—the current will of the patient or the substituted judgement—are not present, reference is made to the best interest. In the case of EE it was necessary, while respecting the autonomy of the person, to verify if there were elements in her history showing her will regarding the health treatments to be guaranteed in the case of a clinical situation such as that determined by a vegetative state.

The events of TMS and EE have contributed to open, in the USA and in Italy, a heated debate on the subject of end of life, urging an intervention by the legislator in order to guide the judge’s choices [14, 15, 26, 27].

The plurality of the involved legal questions and, sometimes, the ideological conditioning have however affected this work, reflecting on a very articulated regulatory framework.

In the case of the USA, the refusal of medical treatment was recognized by the case law, which extended it also to life support treatments. Cures can be refused by patients in a state of incapacity through the reconstruction of the presumed will (as in the case of TMS) or specific declarations.

Eluana Englaro’s case led to the approval, in Italy, after years of debate, of Law No. 219/2017 [28]. This law disciplines different aspects of the legal approach to end-of life issues and it tries to allow both doctors and patients to define the extent, limits and criteria for medical treatments in parallel to constitutional rights and principles set out by the Italian Code of Medical Ethics. The Advance directives (Ads) and the Advance Care Plannings (ACP) are two new tools defined by this law. To briefly describe them, Ads can be seen as a general draft written by a person (healthy) where he/she can also indicate the treatments he/she wishes to undergo or not undergo (in general and/or in case of specific pathologies), and can include the indication of an Health care proxy, who will act on behalf of the patient in relations with doctors. Whereas the ACP can be interpreted as the process in which a patient, by consulting with doctors, relatives and other contacts, decides at what level of intensity and quality of treatment he/she wishes to undergo if he/she becomes incompetent.

The legal approach to the two cases has some common elements: the need to be certain about the diagnosis and prognosis and the fact that the clinical criteria are necessary in determining the most appropriate treatments, although these criteria are not sufficient unless they are supplemented by the patient’s will. Since the patient cannot express himself, and since he has not left written statements, his will must be reconstructed in compliance with the principle of autonomy. This is when the legal representatives take on a decisive role. In Theresa Schiavo’s case, the interpretative difference is between the husband and the patient’s family, while in Eluana’s case it is between the legal representative and the doctors. Interpreting a will to decide whether to suspend a treatment is a complex operation and not a mere procedural act. The issue of relations takes on importance both from a legal and an ethical point of view, but from two different perspectives. While ethics safeguard relationships by guaranteeing their differences and making them reconcilable, law safeguards relationships by guaranteeing the cold forms of respect, equality, impartiality, symmetry, reciprocity, and irreversibility [29].

Like TMS and EE explain us, the role of the human relationships is fundamental both for dignity of life as well as for the system in which a person lives. In this sense, the free therapeutic choice could be re-interpreted. Our identity depends on our relationship with the others, on the culture and traditions in which we live. Relationships with relatives and subsequently with friends give each of us good reason to make use of our personal freedom. Culture indicates which forms of life are worthy of praise in that they are recognized and approved by a certain community in a specific historical context. For this reason, our decisions concern also other people and our community. This link between the self and the other, between “I” and “we”, must be taken into account when making a decision [30]: we certainly decide for ourselves but, given the bond that connects us the others, we also decide for the others. Likewise, when someone else decides for us, they certainly decide for us but also for themselves. None of the involved subjects—patient, family members, health personnel—can be considered neutral: each subject is involved in his/her own identity, in respect of their respective roles [31–33]. Therefore, when it is necessary to reconstruct, as in the two cases that we have analyzed, the will of an individual who is no longer able to express himself/herself—especially, but not only, when there are no written statements—it is necessary to interpret his/her wishes taking his/her relationships into due account [34] Only this hermeneutic work will make statements made at different times and in different contexts univocal. Being a witness does not only imply telling about the other; inevitably one also tells about oneself and about how the bond with others has affected oneself. So, in telling about the other I am telling about myself, as well as in taking care of the other (even the other who does not recognize me and cannot appreciate my care) I am taking care of myself.

### Time of illness and time of care

The third and last point that we want to discuss is related to time of illness and time of care

(WANH) [35, 36]*.* Some important ethical debates ensued following the death of TMS and EE, like those on Aruna Shanbaug in India (a person who has spent 42 years in VS) [37] or those on the recent case of Vincent Lambert in France [28, 29]. In particular, one discussion in which artificial nutrition and hydration were considered medical acts and that the medical administration of fluids and nutrition can be withdrawn if the treatment no longer provides any benefit to the patient [40, 41]. Normally, it is estimated that from the suspension of the artificial nutrition to death a patient spent more or less three days before he/she died [42]. However, this is not a predictable time and the real time spent without fluids and nutrition can be very variable. A Recent article by Fournier et al. [43], showed as WANH applied to babies with terminal pathologies allows the dying process to have some positive features, without being too difficult to endure. They wrote that “*it gives the family extra time to say goodbye, but not so much time that one is reduced to awaiting the infant’s death as a deliverance from torment*”. However, the authors found some interesting data: beyond these 3–4-days, the parents perceived that the waiting time after WANH becomes too long and they felt that the procedure to which their baby was being subjected was cruel and profoundly distressing. Parents began to doubt about severity of the disease and they wondered if their infant would not rather die from starvation than from the disease, which was perceived as unbearable from parents. We found this information important for our discussion, although it is referred to infant patients with terminal pathologies and not adult patients with a chronic condition like DOC, because it seems to underline that the administration of a “time interval” before the death of a patient is crucial for those who care the patient [44–46].

In another sense, we found the “time of illness and care” area important in both cases we analysed. Recalling the role of relationships on people’s identity, as mentioned above, it is necessary to carefully evaluate the role that time, understood as “lived time” (*Kairos*), and not only as “chronological time” (*Kronos*), plays in decision-making, especially when it is necessary to decide for others. This involves recognizing that time has a bearing on the patient's perception of his illness and treatment and, in our case, on that of his family members’ as well and on how it affects their choices [47]. If, as we have already mentioned, making decisions on behalf of someone else calls into question the person who makes the decision, the passing of time (Kronos) affects his identity and changes the meaning of treatment. Emotions are assessed, the tolerability of the burdens is considered, and this makes it possible to understand, over time, to what extent one can endure and when the time has come (kairos) to surrender. There is a time to endure and a time to surrender, both for the patient and for his family [48]. Deciding “when” is a responsibility that each person must take on because, in deciding for the other person, one is deciding for oneself.

### The limits of the analysis

Considering the limits of this study, the first point was that the two cases were separated by 4 years (TMS died in 2005 and EE in 2009) and this should be taken into consideration for the final conclusions. The ethical debate that followed the case of TMS probably made as a background for the Italian judges as well as other debates on case described before TMS as those on Karen Ann Quillan (56) causing different cultural background between the two legal processes in addition to the well-known differences between the American and Italian legal systems. Another limitation is that we focused our analysis on few documents only, excluding the judgements made before by other Courts. However, considering the differences in the legal system of the two States, we preferred to analyse only the final documents about TMS and EE as they occurred more than ten years ago, inferring that the final documents could be seen as the maximum synthesis, which makes them useful for comparing the cases freely available in Italian and English language.

## Conclusions

Both the EE and TMS cases were very important as they have increased the debates on the life-end issue and have encouraged the political debate and the intervention of the legislator in order to guide the judge’s choices. The most interesting element that emerges from our analysis concerns the issue of relationships and how they affect decisions, both from a legal and ethical point of view. In both cases, there were no written statements from the patients which might settle the question regarding the treatments. There was therefore a need for an interpretation which, in our opinion, should evaluate the overall history of these patients by placing their occasional and sporadic statements within the context of their relationships. The fatigue of this hermeneutic practice, which is aimed to understand the meaning of those statements, is the only way to truly respect the autonomy of patients. When, as in our case, conflicts arise on the interpretation given by the various caregivers as to the wishes of patients, it is necessary to recognize the fundamental role of time—understood as lived and not chronological time—spent by these caregivers in caring for  their patients. In deciding for the other, they also decide for themselves. For this reason, during the patients’ hospitalization, it is necessary to identify ways in which they might give their opinion about the moral issues underlying their choices in a serene and constructive way and with the adequate amount of time. The courtroom, albeit necessary in extreme cases, as well as the under-media coverage, certainly do not represent the best condition for all this to happen. The risk, which has become a reality in both cases, is that the wounds and lacerations between people and society become ever wider and more serious, sometimes incurable. This shows that the legal and ethical perspectives represent two different ways of looking at relationships between people.

## Data Availability

In the reference section were reported all the text used in our analysis.

## Abbreviations

ACP: Advance Care Planning
ADs: Advance directives
DOC: Disorders of consciousness
EE: Eluana Englaro Case
TMS: Theresa Marie Schiavo case
UWS: Unresponsive wakefulness syndrome
VS: Vegetative State
WANH: Withdrawal of artificial nutrition and hydration
